# Processing of expository and narrative texts by low- and high-comprehending children

**DOI:** 10.1007/s11145-017-9789-2

**Published:** 2017-10-24

**Authors:** Astrid Kraal, Arnout W. Koornneef, Nadira Saab, Paul W. van den Broek

**Affiliations:** 1 Department of Education and Child Studies, Leiden University, Wassenaarseweg 52, 2333 AK Leiden, The Netherlands; 2 ICLON, Leiden University Graduate School of Teaching, Leiden, The Netherlands

**Keywords:** Reading comprehension, Reading profiles, Strategy use, Low-comprehending readers, Expository texts, Narrative texts

## Abstract

The present study investigated comprehension processes and strategy use of second-grade low- and high-comprehending readers when reading expository and narrative texts for comprehension. Results from think-aloud protocols indicated that text genre affected the way the readers processed the texts. When reading narrative texts they made more text-based and knowledge-based inferences, and when reading expository texts they made more comments and asked more questions, but also made a higher number of invalid knowledge-based inferences. Furthermore, low- and high-comprehending readers did not differ in the patterns of text-processing strategies used: all readers used a variety of comprehension strategies, ranging from literal repetitions to elaborate knowledge-based inferences. There was one exception: for expository texts, low-comprehending readers generated a higher number of inaccurate elaborative and predictive inferences. Finally, the results confirmed and extended prior research by showing that low-comprehending readers can be classified either as readers who construct a limited mental representation that mainly reflects the literal meaning of the text (struggling *paraphrasers*), or as readers who attempt to enrich their mental representation by generating elaborative and predictive inferences (struggling *elaborators*). A similar dichotomy was observed for high-comprehending readers.

## Introduction

Reading is a vital skill in daily life, in work, and in education. At school, a significant amount of the knowledge is transmitted by texts, so to be successful children need to be able to understand and learn from the texts they read (e.g., Slavin, Lake, Chambers, Cheung, & Davis, 2009). Reading comprehension is not only an important skill; it also is a difficult skill for many school-going children to master (e.g., Kuhlemeier et al., 2014; National Center for Education Statistics, 2011). Children who have trouble comprehending texts may suffer the consequences in several domains. These problems can hinder them in learning the required content at school, which may lead to poor results on important tests. In addition, they can lead to low self-efficacy, and even to behavioral problems (e.g., Hall, 2004). Given these far-reaching consequences, it is important that we understand why these children experience problems in reading comprehension.

Central to frameworks on reading comprehension (for an overview see McNamara & Magliano, 2009) is the idea that readers construct a mental text representation of what the text is about (Kintsch, 1988; Trabasso, Secco, & van den Broek, 1984; van den Broek, 1994). Many cognitive processes are involved in the construction of a mental text representation. It calls for basic language skills such as word decoding, syntactic skills, and word knowledge (vocabulary). In addition, successful comprehension also requires higher-level cognitive skills such as inference generation, comprehension monitoring, and knowledge of text structure (Cain & Oakhill, 1999; Oakhill, Cain, & Bryant, 2003; Perfetti, Landi, & Oakhill, 2005; Rapp, van den Broek, McMaster, Kendeou, & Espin, 2007; van den Broek, Rapp, & Kendeou, 2005).

In the current study, we focus on such higher-order cognitive skills. In a think-aloud study we investigate the differences between low- and high-comprehending readers in elementary school (second grade) in terms of their cognitive abilities and of the reading strategies they deploy to comprehend narrative and expository texts. In doing so, we also consider potential differences *within* the low- and high-comprehending reader groups. The goal is to gain insight into the important processes involved in reading comprehension and strategy use at an early age and to identify aspects of reading that hamper text comprehension for less proficient readers in elementary school.

### Reading comprehension processes and strategy use of proficient and struggling readers

It is important to distinguish between comprehension processes and comprehension products. The product is what readers understand and know after reading a text (the mental text representation), whereas the comprehension processes concern the cognitive activities that readers deploy to construct that representation (Rapp et al., 2007). It is commonly assumed that readers (consciously or subconsciously) execute strategies to facilitate comprehension (Pressley & Afflerbach, 1995). Pearson, Roehler, Dole, and Duffy (1992), among others, provided a detailed description of key strategies that are part of the ‘toolkit’ of successful readers. These readers use their general background knowledge and their knowledge of text structure to make sense of texts (Duke & Pearson, 2002; Oakhill & Cain, 2012; Pearson et al., 1992; Pressley & Wharton-McDonald, 1997; Rapp et al., 2007). Successful readers are metacognitive, goal-oriented, and flexible, and they constantly monitor their mental representation (Pearson et al., 1992). They have a clear goal for reading a text (Duke & Pearson, 2002; Pressley & Wharton-McDonald, 1997), try to determine the meaning of unfamiliar words and concepts (Duke & Pearson, 2002), and adjust their reading strategies to the task and the text to deal with inconsistencies and/or information gaps (Duke and Pearson, 2002, Rapp et al., 2007). Successful readers are also well equipped to determine what is important in a text and to summarize and rehearse the information they want to remember (Duke & Pearson, 2002; Pearson et al., 1992; Pressley & Wharton-McDonald, 1997). Moreover, successful readers synthesize information when they read, and draw inferences during and after reading to construct a coherent mental representation of the meaning of the text (Duke & Pearson, 2002; Pearson et al., 1992; Pressley & Wharton-McDonald, 1997; Rapp et al., 2007). Importantly, they make text-connecting inferences by connecting key ideas within the text, and knowledge-based inferences by relating their prior knowledge to these ideas (Perfetti et al., 2005; Pressley & Wharton-McDonald, 1997; Rapp et al., 2007; van den Broek, White, Kendeou, & Carlson, 2009).

Struggling readers also engage in these comprehension processes and strategies, but they tend to do so to a lesser extent or are less adept. For instance, struggling readers often do not use all their relevant prior knowledge to make sense of a story (Pearson et al., 1992) and are less aware of the characteristics of stories (text genre) that might help them in the construction of a mental representation (Oakhill & Cain, 2012; Perfetti et al., 2005). They also are less proficient at comprehension monitoring (e.g., Perfetti et al., 2005) and less likely to adjust their reading when comprehension fails, presumably because they do not possess adequate repair strategies (Pearson et al., 1992). Furthermore, low-comprehending readers are less proficient at judging what is important in texts, and they have difficulties synthesizing information when they read texts with a complex structure (Pearson et al., 1992). Finally, low-comprehending readers may struggle with identifying certain text relations, integrating information from the text with their background knowledge, and generating relevant inferences at the right moments (Cain, Oakhill, Barnes, & Bryant, 2001; Perfetti et al., 2005; Rapp et al., 2007).

However, not all low-comprehending readers struggle with all of these issues, nor do they form a homogeneous group of readers. For example, McMaster et al. (2012) and Rapp et al. (2007) distinguished two types of struggling readers. The first type are labeled *paraphrasers*: they remain close to the literal meaning of the text by rereading or paraphrasing it and make relatively few text-connecting and knowledge-based inferences. *Elaborators,* in contrast, generate more text-connecting inferences and go beyond the text by making knowledge-based inferences–as proficient readers do–but often do not succeed in doing so correctly.

From an educational point of view, it is important to distinguish between subgroups of struggling readers. For example, although the scores of elaborating and paraphrasing struggling readers on after-reading comprehension tests are similarly low (McMaster et al., 2012; Rapp et al., 2007), they seem to benefit from different intervention programs. Elaborators benefit particularly from causal questioning during reading (*“Why…”*), since this helps them to focus on important information within the text. Paraphrasers, on the other hand, benefit particularly from general questioning during reading (“*How does this sentence connect to an earlier part of the text?*”), since such questions prompt them to make more text-based connections and to think about the text beyond the current sentence (McMaster et al., 2012; McMaster, Espin, & van den Broek, 2014).

### The influence of text genre on comprehension processes and strategy use

In the early grades of elementary school the focus of reading instruction lies on technical aspects of learning to read, such as sound-letter correspondences, decoding, and grammar. In later years children read for the purpose of comprehending and learning content from texts on history, geography, science, and other subject areas. In fact, already in fourth grade merely processing the text no longer suffices; children are expected to acquire information from the text for later use (Allington & Johnston, 2002). This shift from *learning to read* to *reading to learn* is accompanied by a change in the type of texts children read at school (Chall, Jacobs, & Baldwin, 1990). Whereas in the early grades children mainly read narrative texts, in later grades expository texts become dominant.

Narrative and expository texts differ in the ways they are structured, the causal coherence of information, the vocabulary, and the presence of a protagonist (Wolfe, 2005). Most children find expository texts more difficult to comprehend than narrative texts (Best, Floyd, & McNamara, 2008), but this is particularly the case for struggling readers (Williams, Hall, & Lauer, 2004). There are several reasons why expository texts pose a challenge. One reason is unfamiliarity: Children are often unfamiliar with expository texts as most reading activities in the early grades in elementary school revolve around narrative texts (Duke, 2000b; Williams et al., 2004). Furthermore, expository texts tend to be more complex than narrative texts, because they often present the children with new (and often abstract) concepts and complex relations, and because their informational density tends to be high (Coté, Goldman, & Saul, 1998; Meyer & Ray, 2011). In addition, expository texts show considerable variability in their local and global structure: They often incorporate a combination of different types of text structures, such as comparison and contrast, cause and effect, problem and solution, and sequence and description (Duke, 2000a; Meyer, 1975, 1985; Williams et al., 2007).

As a result of these fundamental differences between narrative and expository texts, these texts elicit different processing strategies (McDaniel & Einstein, 1989). Expository texts draw more on background knowledge and evoke processing of details, whereas narrative texts elicit processing of the thematic structure and not so much of details (Kintsch & Young, 1984; Wolfe, 2005). Moreover, narratives may evoke more knowledge-based elaborations because children have more background knowledge relevant to the content of narratives than of expository texts. In contrast, expository texts may be processed in a more literal sense and may elicit fewer knowledge-based elaborations (Coté et al., 1998).

### The present study

Prior research on comprehension processes and on-line strategy use of young children has focused on the comprehension of narrative texts (e.g., Cain et al., 2001; Cain & Oakhill, 1999; Cain, Oakhill, & Bryant, 2004; Kendeou et al., 2005). Research on the comprehension of expository texts in the early grades of elementary school (first and second grades) is particularly scarce (but see e.g., Duke, Bennett-Armistead, & Roberts, 2002, 2003; Williams et al., 2004). Addressing this gap, the present study aims to investigate young children’s processing and strategy use, in particular with respect to expository texts. Specific questions are whether the distinction between paraphrasers and elaborators already exists at a very young age, and whether processing patterns apply to expository as well as narrative texts. These issues were investigated in a think-aloud study in which second-grade pupils^1^ read narrative and expository texts in a sentence-by-sentence manner and were asked to express their thoughts after each sentence. To assess the quality of their after-reading mental text representation, we asked the children literal and inferential (text-connecting and knowledge-based) comprehension questions (Cain & Oakhill, 1999). To determine possible factors contributing to comprehension differences, we also had them complete a test battery assessing general cognitive and language-related proficiencies.

## Method

### Participants

The study included 87 second-grade pupils (51 girls; mean age 7:8, range 7:2–8:7) from 19 classes of nine public elementary schools in the Netherlands, ranging from rural to inner-city schools. They were selected from a larger screening sample (N = 385) on the basis of the following inclusion criteria: (1) average or above-average scores on a non-verbal intelligence test (*Raven’s progressive matrices*, Raven, Raven, & Court, 1998); (2) average or above-average scores on a Dutch standardized test for word reading ability (*DMT* [Three Minute Test], Cito, 2009); (3) no diagnosed behavioral and/or attention problems. On the basis of their scores on a Dutch standardized test for reading comprehension (*LOVS Begrijpend Lezen Groep 3* [Reading comprehension test for Grade 1], Cito, 2006), the selected children were assigned to two groups: high-comprehending readers (*N* = 57; > 75th percentile) and low-comprehending readers (*N* = 30; < 50th percentile). Before testing, the parents or guardians signed a letter of active consent. After testing, the children received an eraser, and their teachers received a book token (€20).

### Measures and materials

#### Think-aloud session: texts and questions

In the think-aloud session participants read two expository and two narrative texts. The four experimental texts were matched on readability and length.^2^ The sentences of the texts were printed in font Arial, font size 12 on cards of 10 × 15 cm. The cards were presented in flip-over photo albums. A practice text was presented in a separate photo album. Text comprehension was assessed by posing five questions after each text.

Before the test session started, an examiner explained the think-aloud procedure to the child, and modeled it by reading part of the practice text. The child practiced the procedure on the remainder of the practice text. Think-aloud responses were audio-recorded.

#### Pre-processing of think-aloud data

The recordings of the verbal protocols were transcribed and parsed into idea units (see for details Tabasso & van den Broek, 1985) by trained assistants. First, three raters parsed nine transcripts independently (inter-rater reliability K = .87). Subsequently, two raters parsed the remaining transcripts. Problematic cases were resolved through discussions.

After this initial parsing procedure the idea units were coded into eight categories using coding sheets based on guidelines by Linderholm and van den Broek (2002), McMaster et al. (2012), and Rapp et al. (2007). The category *Restating the Sentence* includes text repetitions and paraphrases, meaning that the reader restates the text verbatim or rephrases a sentence in his/her own words. *Explaining the Sentence* indicates that the reader provides an explanation for the contents of the current sentence by connecting its meaning to the preceding text. We speak of an *inference* when the reader provides an explanation for the contents of the current sentence on the basis of background knowledge (*Elaborative Inference)* or anticipates or predicts what will occur next in the text *(Predictive Inference)*. These elaborative and predictive inferences can be characterized as either *valid* or *invalid* in the context of the text. The category *Comments* includes associations, affective responses, evaluative comments, and metacognitive comments by the reader. The category *Question* applies when the reader asks or implies a question about the content of the text. *Silent Period* refers to the situation when the reader does not verbalize his or her thoughts for the space of 3 s or longer. *Other* is a miscellaneous category that includes all other responses, as well as passages that are inaudible. Using this procedure of coding the responses, three independent raters coded 15% of the transcripts, resulting in an inter-rater reliability score of 66% and an average correlation of *r* = .85. Two independent raters coded the remaining transcripts. Disagreements were resolved by discussion.

Only the first categorized idea unit of the responses for each sentence was used in the analyses. The reason for this was that the first response is the most spontaneous, and this procedure results in equal numbers of responses for all participants, thereby making comparisons between participants possible, see Ericsson and Simon, (1980, 1993).

### Test battery

#### Non-verbal intelligence

Raven’s Standard Progressive Matrices (Raven, Raven, & Court, 1998) was used as a measure for non-verbal intelligence and abstract reasoning. Reported scores are raw scores with a maximum possible score of 60.

#### Word reading ability

A Dutch standardized test was applied (the ‘Drie-Minuten-Toets’—DMT, *3*-*min test*—Cito, 2009) to assess word decoding skills. Within 1 min, children read aloud as many words as possible. The test had been administered by the schools at the end of Grade 1. Reported scores are skills scores.

#### Reading comprehension

A Dutch standardized test (Cito Leerling- en onderwijsvolgsysteem Begrijpend Lezen Groep 3—*Cito Reading Comprehension Test Grade 1*—Cito, 2006) was used to assess reading comprehension. The test consists of three modules: an initial module for all children, an easier follow-up module for weak comprehenders, and a more difficult follow-up module for average and good comprehenders. In six of the nine schools, the children had taken this test at the end of Grade 1. At three schools, we administered the initial module of the test ourselves. Reported scores are skill scores.

#### Listening comprehension

A standardized Dutch test was used to assess listening comprehension (Cito Begrijpend Luisteren 1 & 2—*Cito, Comprehensive Listening 1 & 2*—Cito, 2011). The test consists of two parts. In both parts, children listen to one- to four-sentence stories and answer a question by choosing the right picture from three pictures. Reported scores are skill scores.

#### Vocabulary knowledge

The Peabody Picture Vocabulary Test-III-NL (Schlichting, 2005) was used as a standardized measure to assess receptive vocabulary in Dutch. The test consists of words ranging in difficulty. Each word is presented with four pictures, one of which represents the word. Reported scores are raw scores (maximum score of 60).

#### Reading skills

We developed a Maze test (Espin, Busch, & Shin, 2001) consisting of two experimental texts (and one practice text) in which every sixth word was replaced by a blank. Children filled in the blanks by identifying the correct word out of three options. There was a 2-min time limit for each text. Reported scores are raw scores (maximum score of 37).

#### Story structure recognition

We translated the Story Anagram Task (Oakhill et al., 2003). The test consists of four six-sentence stories that are cut up to single sentences and displayed to the participants in a random order. Participants are asked to arrange the sentences in the correct order. In the original scoring procedure, participants receive one point for each sentence that is put in the correct order. We adopted a more liberal scoring procedure and assigned points to correct combinations of sentences as well. Reported scores are alternative raw scores (maximum score of 28).

#### Inference making

We translated the Inference and Integration Task (Cain & Oakhill, 1999). The test consists of three test stories with six comprehension questions each: two questions tapping literal information, two questions requiring a text-connecting inference, and two questions requiring a gap-filling inference. Reported scores are raw scores (maximum score of 18).

#### Verbal working memory

We translated and adapted the Sentence Span Measure (Swanson, Cochran, & Ewers, 1989). The original test consists of four levels with two sets of unrelated declarative sentences with levels increasing in difficulty: the lowest level consists of two sets of two sentences, the highest level of two sets of five sentences. We added an easier level consisting of two sets of one sentence, because in a pilot test the original lowest level (two sets of two sentences) proved to be too difficult for many children. As in the original test, the sentences were seven to ten words in length. Reported scores are the scores for number of words correctly remembered plus comprehension questions correctly answered.

#### Socioeconomic status

We translated the Family Affluence Scale (FAS) (Currie, Elton, Todd, & Platt, 1997) to assess the socioeconomic status of the children. Reported scores are raw scores (maximum score of 9).

#### Reading motivation and reading attitude

We translated the Elementary Reading Attitude Survey (ERAS) (McKenna & Kear, 1990) to assess reading motivation and attitude. The survey consists of statements referring to school-related reading and recreational reading. Reported scores are raw scores (maximum score of 80).

### Procedure

Participants were tested in five sessions. The first three (group-administered) sessions took place, at one-week-intervals, at the beginning of Grade 2. In the first session the children completed the assessment for socioeconomic status, part I of the listening comprehension test, and the assessment for reading motivation. In the second session, the children completed part II of the listening comprehension test, the assessment for receptive vocabulary, and the Maze test for reading skills. In the third session, the children completed the assessments for non-verbal intelligence and the standardized test for reading comprehension if this test had not previously been administered by the school. A session lasted no longer than 60 min.

In the two remaining sessions, the children—who had by then spent 6 months in Grade 2—were tested individually. In the first individual session (30 min), the children completed the test battery—i.e., story structure recognition, the ability to make inferences, and verbal working memory capacity were assessed. In the second individual session (45 min), the think-aloud protocol was employed.

## Results

### Test battery

Data of one low-comprehending participant were missing on all the tests of the test battery; data of one low-comprehending participant were missing for the tests on reading skills and vocabulary knowledge; data of one high-comprehending participant were missing for the test on verbal working memory. Fifteen participants (3 low- and 12 high-comprehending readers) only completed the initial part of the reading comprehension test.

Independent-samples *t* tests revealed that the group of high-comprehending readers outperformed the group of low-comprehending readers on all tasks in the test battery, with the exception of the test on reading motivation (see Table 1).

**Table 1:** Results of low-comprehending readers and high-comprehending readers on tasks in the test battery

Measure	Low-comprehending readers	High-comprehending readers	Levene’s test (*p* value)	*t* value	*df*	*p* value
	M (SD)	M (SD)				
Reading comprehension	− 7.85 (6.44)	19.02 (7.49)	.122	− 15.31	69	< .001
Word reading ability	45.31 (9.45)	54.88 (12.62)	.057	− 3.60	84	.001
Non-verbal intelligence	27.90 (7.24)	35.30 (6.80)	.489	− 4.67	84	< .001
Listening comprehension	49.93 (9.11)	60.86 (8.30)	.371	− 5.59	84	< .001
Socioeconomic status	5.79 (2.21)	7.19 (1.27)	.001	− 3.16	37.727	.003
Reading skills	19.46 (5.85)	25.32 (7.11)	.128	− 3.77	83	< .001
Vocabulary knowledge	33.61 (6.18)	41.65 (6.15)	.895	− 5.66	83	< .001
Reading motivation	64.45 (12.30)	60.62 (10.57)	.479	1.50	84	.137
Story structure recognition	18.83 (4.94)	23.12 (4.01)	.254	− 4.34	84	< .001
Inference making	12.67 (2.29)	14.68 (1.42)	.011	− 4.30	39.196	< .001
Verbal working memory	2.83 (1.14)	5.68 (3.25)	.000	− 5.90	75.704	< .001

### Think-aloud experiment

#### Processing strategies of low-comprehending and high-comprehending readers

The data for the think-aloud experiment were analyzed in a multivariate Repeated Measures (RM) ANOVA, with Text Genre (narrative vs. expository texts) as within-participant factor, and Reading Proficiency (high-comprehending vs. low-comprehending readers) as between-participant factor. The dependent variables were the percentages of each of the strategy categories. The analyses revealed a significant main effect of Text Genre (*F*(7.79) = 15.14, *p* < .001) and a marginally significant main effect of Reading Proficiency (*F*(7.79) = 1.88, *p* = .084).

**Fig. 1:**
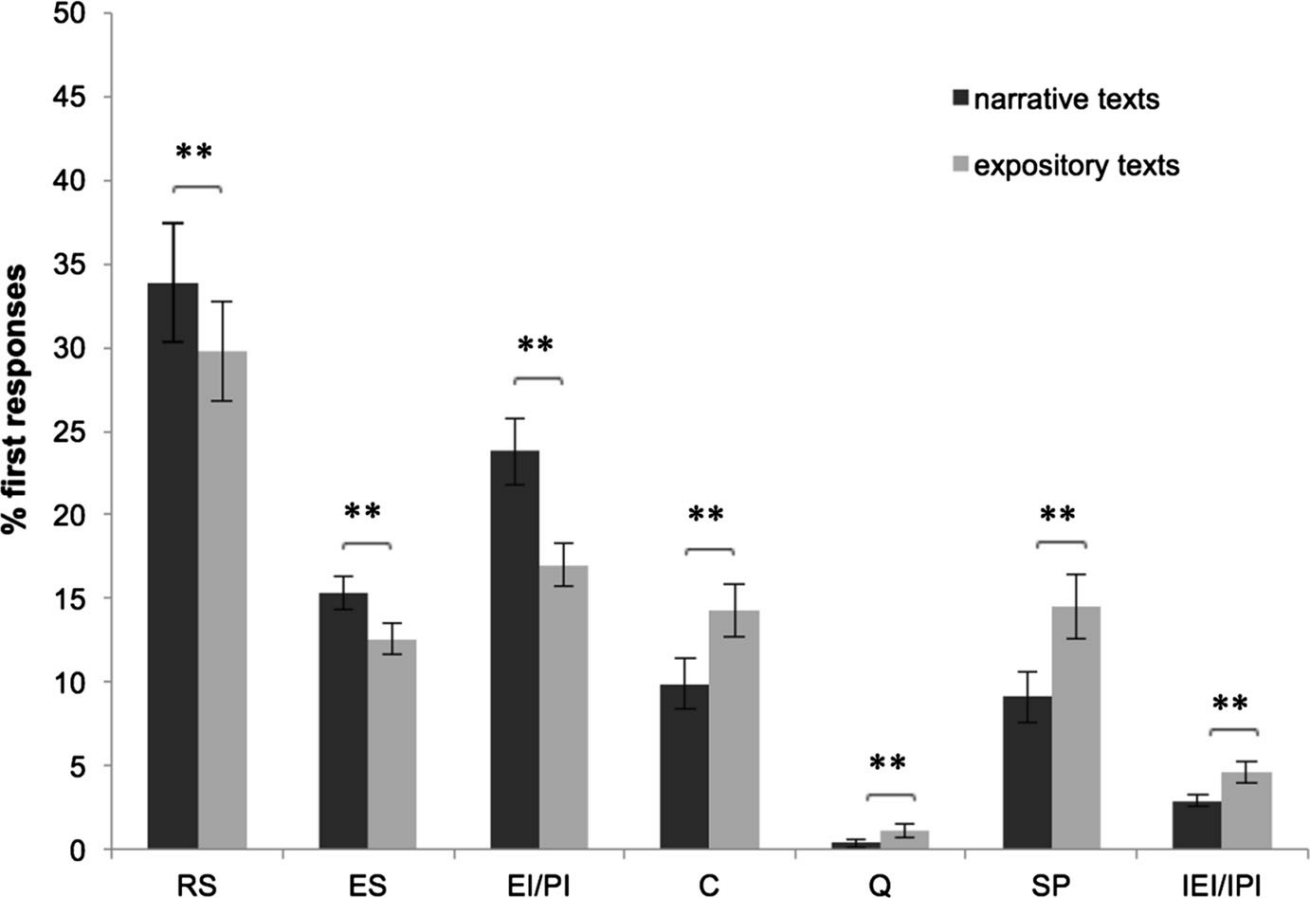
Mean percentages of first responses on the strategy categories for narrative and expository texts. *RS* restating the sentence, *ES* explaining the sentence, *EI/PI* elaborative/predictive inference, *C* comment, *Q* question, *SP* silent period, *IEI/IPI* invalid elaborative/predictive inference. **p < .01

Univariate ANOVAs revealed that the main effect of Text Genre was present in all response categories (see Fig. 1). Response categories that were observed more often in narrative texts than in expository texts were Restating the Sentence (*F*(1.85) = 9.188, *p* = .003), Explaining the Sentence (*F*(1.85) = 11.763, *p* = .001), and Valid Inferences (*F*(1.85) = 16.278, *p* < .001). The response categories that were observed more often for expository texts were Comments *F*(1.85) = 16.795, *p* < .001), Question *F*(1.85) = 8.813, *p* = .004), Silent Period *F*(1.85) = 35.668, *p* < .001), and Invalid Inferences *F*(1.85) = 14.106, *p* < .001).

In addition, the univariate ANOVAs revealed an interaction effect between Text Genre and Reading Proficiency for the category Invalid Inferences (*F*(1.85) = 5.10, *p* = .026). Post-hoc independent-samples *t* tests indicated that low-comprehending readers made more invalid inferences than high-comprehending readers when they were reading expository texts (*t*(34.27) = 2.13, *p* = .04). This contrast between the two groups was absent for the narrative texts (t(42.84) = 1.05, *p* = .30).

#### Text representation of low-comprehending and high-comprehending readers

**Fig. 2:**
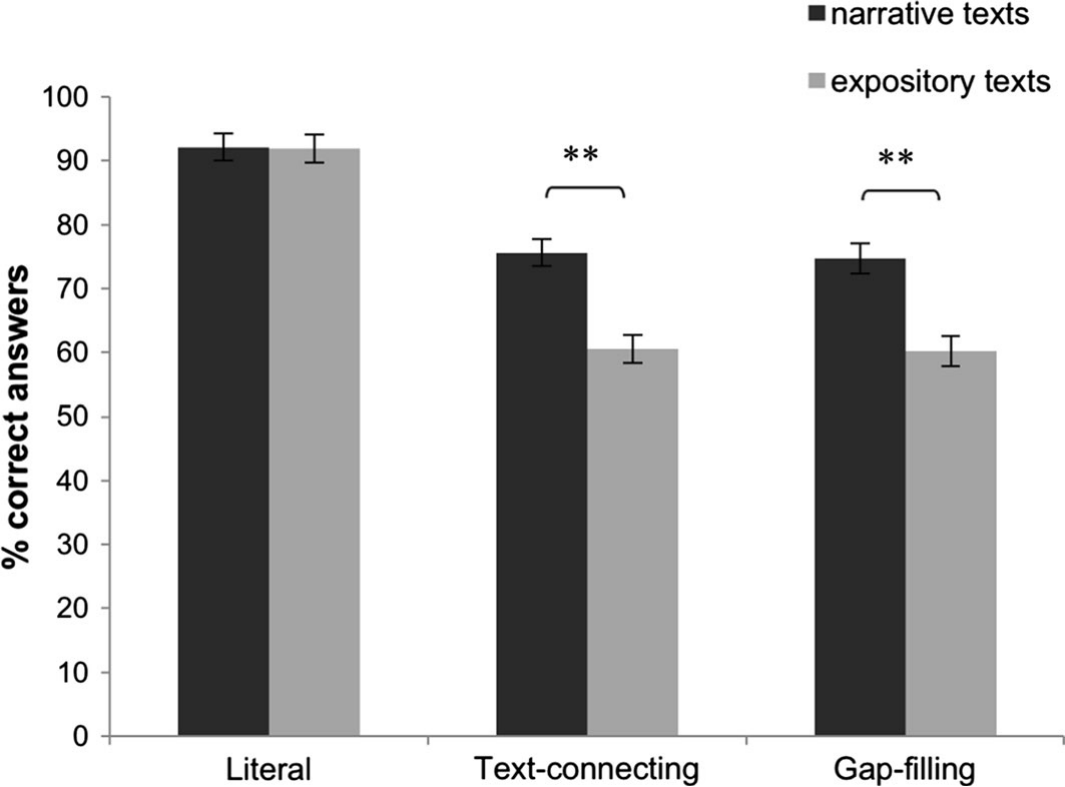
Percentages of mean correct responses on comprehension questions for narrative and expository texts. **p < .01

Data of one low-comprehending participant were missing. Performance on the comprehension questions was analyzed with a RM-ANOVA, including the within-participant factors Type of Question (three levels: literal, text-connecting inference, gap-filling inference) and Text Genre, and the between-participant factor Reading Proficiency. The dependent variable was the percentage of questions answered correctly. The results showed a main effect of Reading Proficiency (*F*(1.84) = 37.64, *p* < .001), indicating that the high-comprehending readers (M = 80.19%, SD = 1.22) outperformed the low-comprehending readers (M = 67.34%, SD = 1.70). A main effect of Text Genre (*F*(1.84) = 32.64, *p* < .001) showed that the participants answered more questions correctly in relation to narrative texts (M = 78.72%, SD = 1.44) than in relation to expository texts (M = 68.82%, SD = 1.27). Also, a main effect of Type of Question was observed (*F*(2168) = 99.05, *p* < .001). Post-hoc paired-samples *t* tests showed that the participants performed better on literal questions (M = 90.16%, SD = 1.32) than on text-connecting questions (M = 65.83%, SD = 1.65), (t(85) = 14.06, *p* < .001), and that they performed better on literal questions than on gap-filling questions (M = 65.31%, SD = 1.71), (t(85) = 11.71, *p* < .001). There was no difference between participants’ performance on text-connecting and gap-filling questions (t(85) = − .65, *p* = .515). In addition, the analysis revealed an interaction effect for Type of Question and Text Genre (*F*(1.84) = 8.29, *p* < .001). Post-hoc paired-samples *t* tests indicated that Text Genre had no effect on participants’ performance on literal questions (*t*(85) = .09, *p* = .929), but that participants performed better on text-connecting and gap-filling questions in relation to narrative texts than in relation to expository texts (text-connecting: *t*(85) = 5.81, *p* < .001; gap-filling: *t*(85) = 4.65, *p* < .001), see Fig. 2.

### Identifying subgroups of readers

#### Subgroups of low-comprehending readers

A cluster analysis was conducted on the group of low-comprehending readers to explore the existence of subcategories. Following McMaster et al. (2012), we used Ward and Hook’s (1963) procedure which aims to minimize the sums of squares of observations within any two clusters that are formed at each step. This procedure revealed two distinct subgroups of low-comprehending readers. To classify these subgroups, a multivariate RM-ANOVA was conducted to examine the distribution of the first responses in the think-aloud experiment, with Text Genre as within-participant factor and Cluster (i.e., the two subgroups of low-comprehending readers) as between-participant factor.

**Fig. 3:**
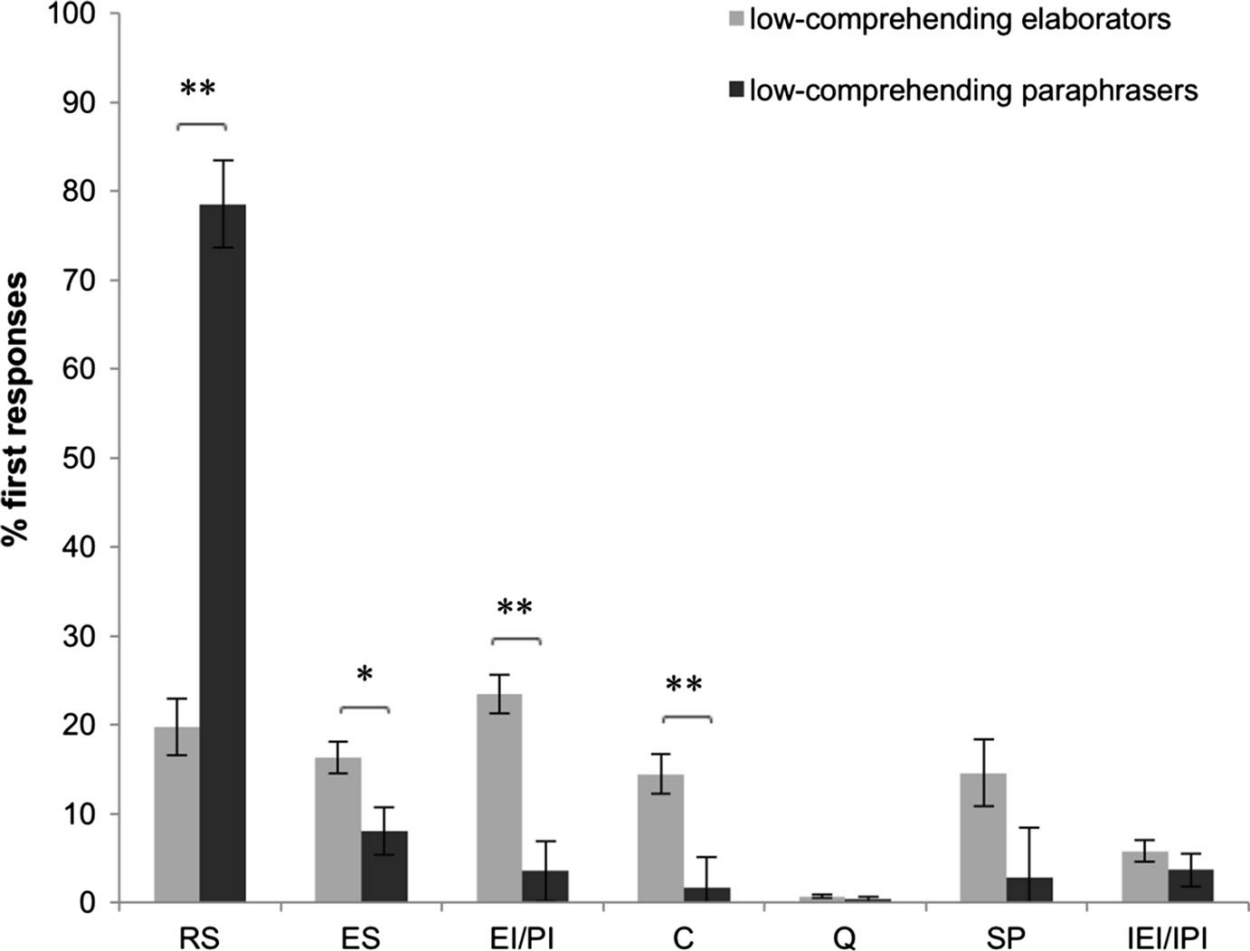
Mean percentages of first responses per subgroup of low-comprehending readers on the strategy categories. *RS* restating the sentence, *ES* explaining the sentence, *EI/PI* elaborative/predictive inference, *C* comment, *Q* question, *SP* silent period, *IEI/IPI* invalid elaborative/predictive inference. *p < .05; ** *p* < .01

The results showed a main effect of Cluster (*F*(7.22) = 14.94, *p* < .001). See Fig. 3. The follow-up analyses revealed that subgroup A of low-comprehending readers (*N* = 9) more frequently displayed think-aloud responses of the type Restating the Sentence than did subgroup B (*F*(1.28) = 98.99, *p* < .001), whereas subgroup B (*N* = 21) more frequently displayed think-aloud responses of the types Explaining the Sentence (*F*(1.28) = 6.45, *p* = .017), Valid Inferences (*F*(1.28) = 24.28, *p* < .001), and Comments (*F*(1.28) = 9.45, *p* < .005). Readers in subgroup A predominantly responded by restating the sentences they read. Therefore, we denote readers in subgroup A as *paraphrasers*. In contrast, responses by readers in subgroup B frequently belonged to categories that involve inference making. We therefore denote readers in subgroup B as *elaborators.*

#### Text representation of elaborating and paraphrasing low-comprehending readers

**Fig. 4:**
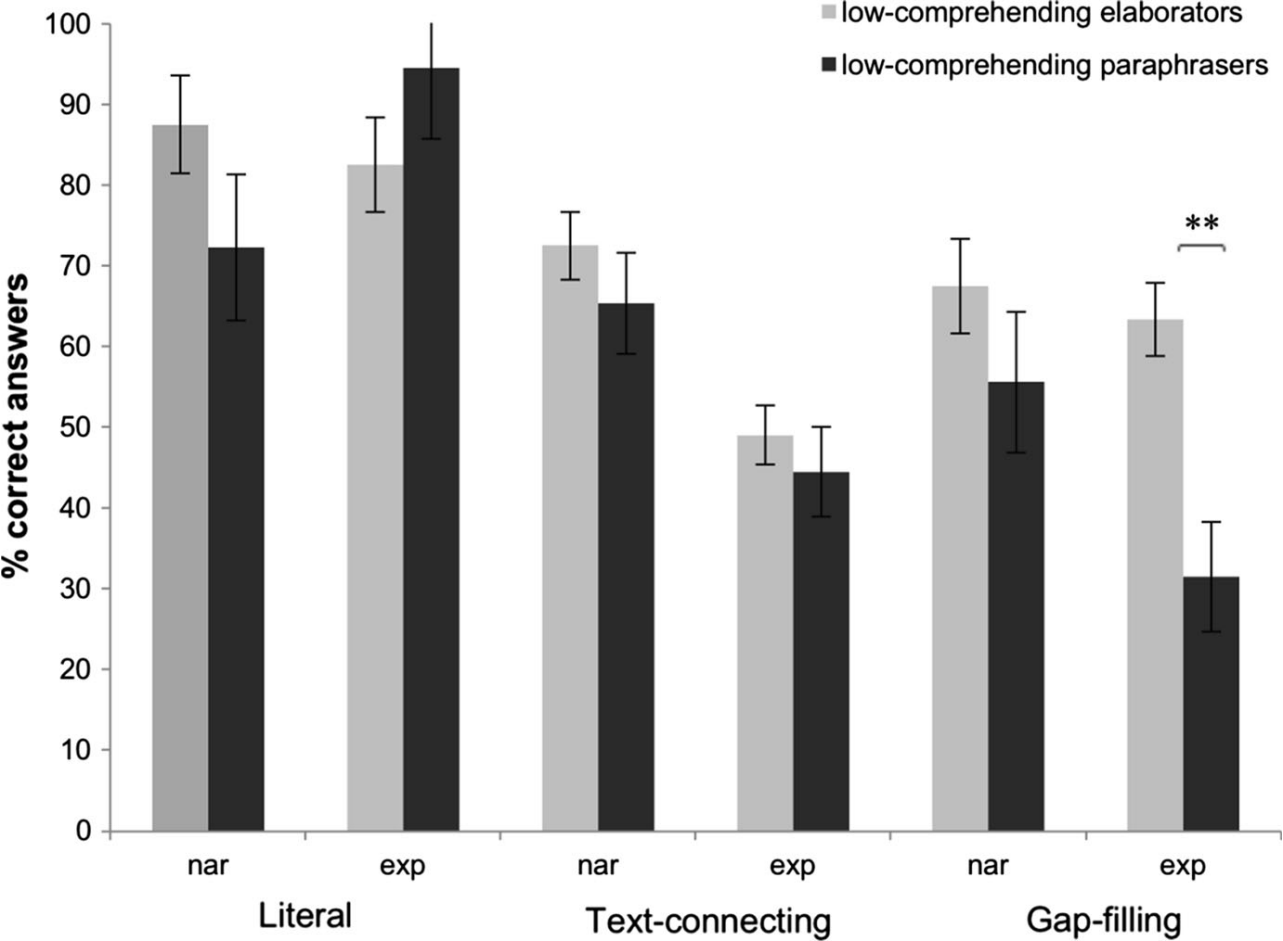
Percentages of mean correct responses per subgroup of low-comprehending readers on comprehension questions for narrative and expository texts. *Nar* narrative texts, *exp* expository texts. **p < .01

We repeated the analyses of the comprehension questions for the subgroups of elaborating and paraphrasing low-comprehending readers to investigate possible differences with respect to text representation. A main effect of Cluster (F(1.27) = 5.44, *p* = .027), showed that elaborators had a higher percentage correct responses (M = 66.75%, SD = 10.07) than paraphrasers (M = 56.67%, SD = 12.50). In addition, a two-way interaction between Type of Question and Cluster was observed (F(2.54 = 3.25, *p* = .046). Post-hoc independent-samples *t* tests revealed that elaborators were better at answering gap-filling questions than paraphrasers (t(27) = 2.79, *p* = .010). This effect was absent for the other types of questions (literal questions: t(27) = 0.26, *p* = .795); text-connecting questions: t(27) = 1.01, *p* = .323). The three-way interaction between Text Genre, Type of Question, and Cluster was also significant (F(2.26) = 3.36, *p* = .042). Post-hoc testing revealed that elaborators were better at answering gap-filling questions about expository texts than paraphrasers (t(27) = 3.91, *p* = .001). This contrast between subgroups was absent for narratives texts (t(27) = 1.13, *p* = .267) (see Fig. 4).

#### Cognitive profiles of low-comprehending elaborators and paraphrasers

Independent-samples *t* tests were conducted to uncover potential differences in the (cognitive) profiles of the two types of low-comprehending readers. These exploratory analyses revealed that elaborators had a higher socioeconomic status than paraphrasers (elaborators: M = 6.5, SD = 1.93, paraphrasers: M = 4.22, SD = 2.05), (t(27) = 2.884, *p* = .008) and that they outperformed paraphrasers on receptive vocabulary knowledge (elaborators: M = 35.37, SD = 5.46, paraphrasers: M = 29.89, SD = 6.23), (t(26) = 2.372, *p* = .025), inference and integration skills (elaborators: M = 13.40, SD = 1.92, paraphrasers: M = 11.06, SD = 2.34), (t(27) = 2.849, *p* = .008), and verbal working-memory capacity (elaborators: M = 3.20, SD = 1.06, paraphrasers: M = 2.00, SD = 0.87), (t(27) = 2.979, *p* = .006). Paraphrasers, in contrast, outperformed elaborators on the test for technical word-reading skills (elaborators: M = 42.45, SD = 7.78, paraphrasers: M = 51.67, SD = 10.17), (t(27) = − 2.685, *p* = .012) and on the Maze test for reading skills (elaborators: M = 17.95, SD = 5.75, paraphrasers: M = 22.67, SD = 4.92), (t(26) = − 2.117, *p* = .044).

#### Subgroups of high-comprehending readers

**Fig. 5:**
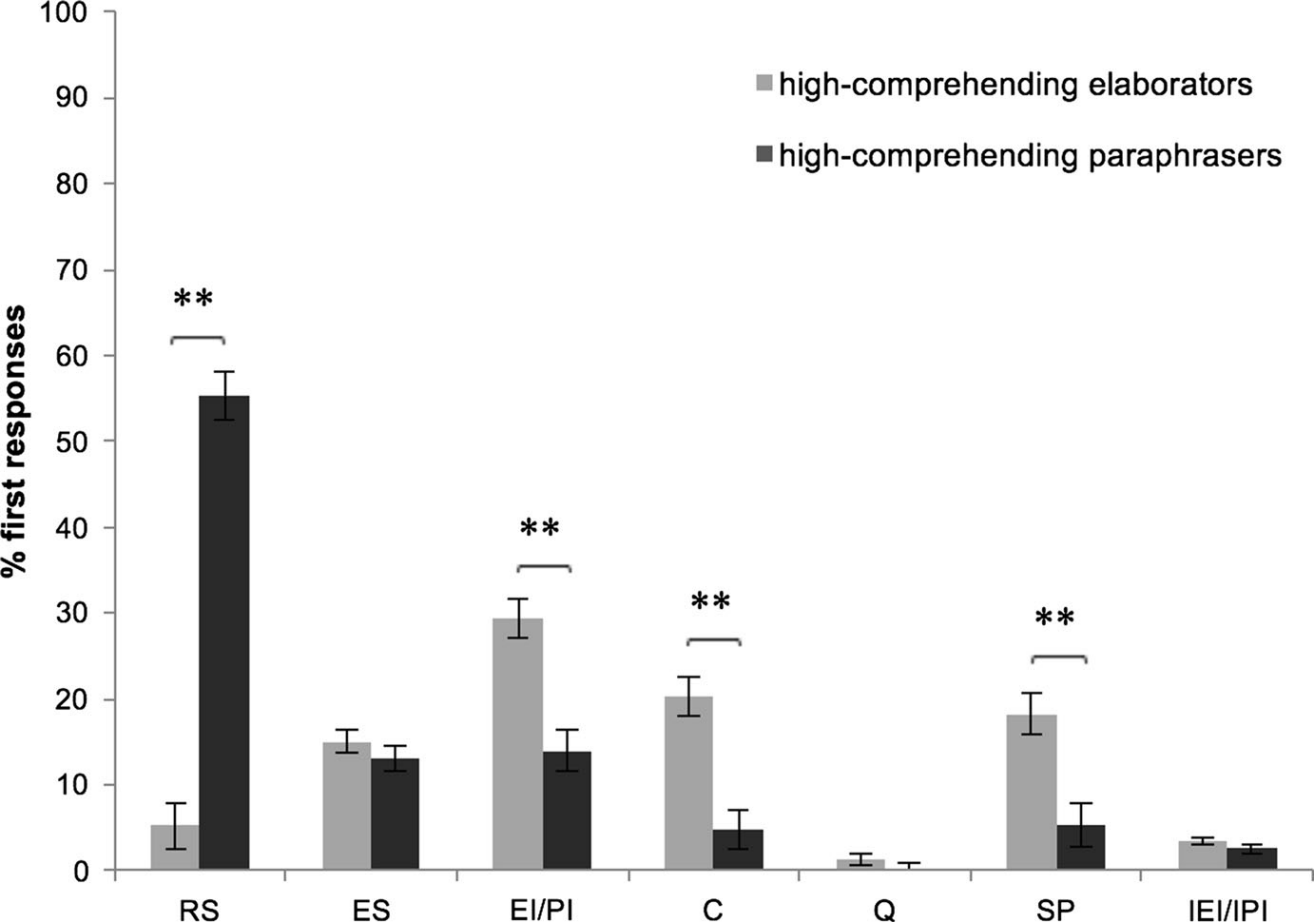
Mean percentages of first responses per subgroup of high-comprehending readers on the strategy categories. *RS* restating the sentence, *ES* explaining the sentence, *EI/PI* elaborative/predictive inference, *C* comment, *Q* question, *SP* silent period, *IEI/IPI* invalid elaborative/predictive inference. **p < .01

We explored the composition of the group of high-comprehending readers with the same cluster procedure as conducted for the low-comprehending readers. The analysis revealed two subgroups of high-comprehending readers. The multivariate RM-ANOVA to classify these subgroups showed a main effect of Cluster (*F*(7.49) = 28.16, *p* < .001), and a two-way interaction for Cluster and Text Genre (*F*(7.49) = 3.18, *p* = .007). Post-hoc analyses revealed that subgroup A (*N* = 27) more frequently displayed think-aloud responses of the type Restating the Sentence (*F*(1.55) = 164.46, *p* < .001) than did subgroup B, whereas subgroup B (*N* = 30) more frequently displayed think-aloud responses of the type Valid Inferences (*F*(1.55) = 20.99, *p* < .001), Comments (*F*(1.55) = 21.65, *p* < .001), and Silent Period (*F*(1.55) = 13.91, *p* < .001) than did subgroup A (see Fig. 5). This mirrors the pattern that we observed for the low-comprehending readers. Readers in subgroup A predominantly responded by restating the sentences they had read. Therefore, we denote readers in subgroup A as *(high*-*comprehending) paraphrasers*. In contrast, responses by readers in subgroup B frequently belonged to categories that involve inference-making. We therefore denote readers in subgroup B as *(high*-*comprehending) elaborators.*

**Fig. 6:**
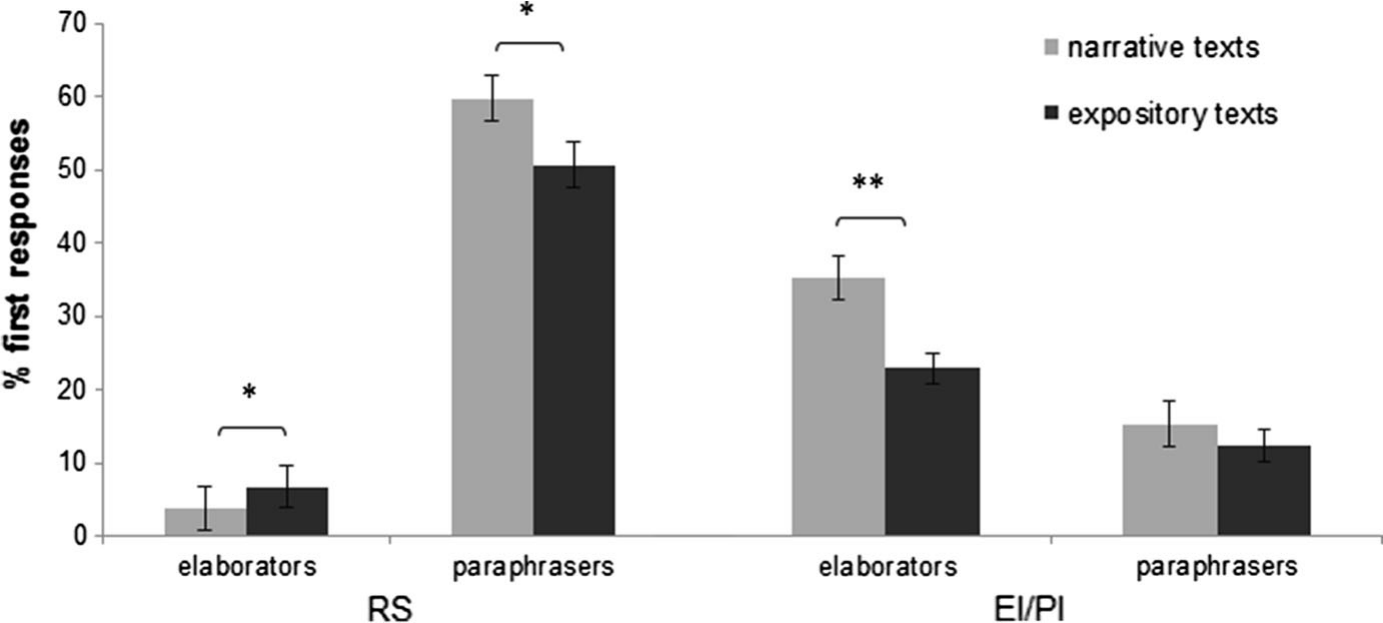
Mean percentages of first responses per subgroup of high-comprehending readers on two strategy categories for narrative and expository texts*. RS* restating the sentence, *EI/PI* elaborative/predictive inference. *p < .05; **p < .01

In addition to the main effect of Cluster, a two-way interaction for Cluster and Text Genre (*F*(7.49) = 3.18, *p* = .007) was observed for two response categories: Restating the Sentence (*F*(1.55) = 11.04, *p* = .002) and Valid Inferences (*F*(1.55) = 7.47, *p* = .008). Paraphrasers repeated or rephrased the sentence more often when reading narrative texts than when reading expository texts (t(26) = 2.56, *p* = .017), whereas elaborators repeated or rephrased the sentence more often when reading expository texts than when reading narrative texts (t(29) = − 2.29, *p* = .030). Text Genre did not influence the responses of paraphrasers on the category Valid Inferences (t(26) = 1.21, *p* = .237), but elaborators made more valid elaborative and predictive inferences when reading narrative texts than when reading expository texts (t(29) = 4.87, *p* < .001) (see Fig. 6).

#### Text representation and cognitive profiles of elaborating and paraphrasing high-comprehending readers

With respect to the comprehension questions, a RM-ANOVA revealed no main or interaction effects of Cluster (*p*’s > .3). This suggests that the subgroups of high-comprehending readers did not differ in their ability to answer the comprehension questions in the think-aloud experiment. This equivalence between subgroups was reflected in their cognitive profiles, as independent samples *t* tests revealed no differences on any of the tasks administered in the test battery (*p*’s > .1).

## Discussion

In this study, we investigated the comprehension processes and strategy use of second-grade low- and high-comprehending readers when reading narrative and expository texts for comprehension. The main findings are as follows. First, the high-comprehending readers performed better than the low-comprehending readers on the comprehension questions posed after each text. Second, while thinking out aloud the children used a variety of comprehension strategies, ranging from textual repetitions and paraphrases to elaborate text-based and knowledge-based inferences. With one exception, there was no evidence that low- and high-comprehending readers differed in their patterns of text-processing strategies, the exception being that for expository texts low-comprehending readers generated more inaccurate inferences than high-comprehending readers. Third, our study showed that young, eight-year-old low-comprehending readers can be classified either as readers who construct a mental representation that emphasizes the literal meaning of the text (paraphrasers) or as readers who embellish their mental representation by generating elaborative and predictive inferences (elaborators). Fourth, we observed a similar division of paraphrasers and elaborators in our group of high-comprehending readers. Finally, our study indicated that text genre affected the way children processed the texts.

### Processing strategies and text comprehension of low- and high-comprehending readers

A robust finding in our study was that high-comprehending readers performed better than low-comprehending readers on the after-reading comprehension questions, for both narrative and expository texts. Concerning the expository texts the think-aloud responses present a relatively straightforward explanation. For these texts, low-comprehending readers generated more inaccurate inferences than did high-comprehending readers. Inclusion of inaccurate inferences had a negative impact on the quality of their overall mental representation of the text. As a result, they performed worse than high-comprehending readers on questions across the board. However, this explanation does not hold for the narrative texts. For narratives, the low-comprehending readers were again outperformed by the high-comprehending readers on all question types, but here their poorer performance was not due to making more inaccurate inferences. Indeed, the low- and high-comprehending readers did not differ in their overall patterns of strategy use. These results should be interpreted with some caution as methodological aspects of the current study may have contributed to the patterns observed. For example, the single-sentence presentation format may have impaired comprehension processes and may have discouraged the use of certain strategies during reading (Coté et al., 1998; Rapp & Mensink, 2011), perhaps differentially for the low and high comprehenders. Likewise, the think-aloud procedure may have influenced the results (Rapp et al., 2007). Therefore, it is important to explore whether these results can be replicated by using different methods. Nevertheless, it is worth speculating about possible explanations for the paradoxical findings concerning narrative texts.

One approach is to assume that although low- and high-comprehending readers do not process narrative texts differently, low-comprehending readers nonetheless experience problems in constructing their mental representation. Rapp et al. (2007) argued that high- and low-comprehending readers possess a similar “toolkit of strategies”, but low-comprehending readers are less likely to use the toolkit effectively. They are either less efficient at using the right tools at the right time (Cain et al., 2001; Perfetti et al., 2005; Rapp et al., 2007), or they use the right tools at the right time, but apply these tools in the wrong way (Pearson et al., 1992; Rapp et al., 2007). Furthermore, it is also possible that low-comprehending readers activate the right type of information by using the right tool at the right time, but that they are still unable to produce a fully coherent mental representation of a text at the global level (Cain et al., 2001). In other words, their micro- and local-processing skills (e.g., their ability to make connections between sentences) are sufficient to establish basic comprehension, but their macro-processing skills (ability to form a mental representation of the text as a whole) are insufficient (Van Dijk & Kintsch, 1983).

A different approach is to relate the inferior quality of low-comprehenders’ text representation to processes of memory retrieval and maintenance. In the current study, the quality of the children’s mental text representations was measured by after-reading comprehension questions. Hence, whether the children were able to answer the questions satisfactorily depended not only on the quality of the mental model they developed as they were reading the text, but also on how proficient they were at keeping the representation active over time, and how easily they retrieved the relevant information from this representation. It is thus possible that the problems of low-comprehending readers did not lie in them forming an inferior initial mental representation, but rather that, due to processes of memory decay and interference, this representation deteriorated more quickly and was more difficult for them to access.

The results of the test battery revealed that in comparison to high-comprehending readers, low-comprehending readers displayed deficiencies in several (cognitive) domains—including decoding skills, higher-level comprehension skills, and verbal working memory capacity—that are closely associated with successful reading comprehension. As a result, it is not feasible to decide which of the alternatives discussed above (i.e., wrong inferences vs. wrong timing of inferences vs. macro-processing deficiencies vs. memory maintenance and retrieval deficiencies) is the most likely underlying cause of the comprehension difficulties. Instead, the most plausible conclusion is that the challenges faced by low-comprehending readers are of a ‘multimorbid’ nature, meaning that some text comprehension deficiencies (and their precursors) exist independently of each other, whereas other deficiencies are intrinsically intertwined (e.g., since they share a common source).

### Subgroups of low- and high-comprehending readers

In line with previous studies (McMaster et al., 2012; Rapp et al., 2007), our study showed that low-comprehending readers can be classified either as struggling paraphrasers or as struggling elaborators. While thinking aloud, the paraphrasers predominantly repeated or paraphrased the sentences of the texts. By contrast, elaborators frequently generated text-based and knowledge-based inferences. For narrative texts, these different processing strategies did not produce reliable differences in performance on the after-reading comprehension questions. This is consistent with the proposal of McMaster et al. (2012), who argued that paraphrasers and elaborators struggle with narrative texts to the same extent, yet for different reasons. Whereas paraphrasers fail to generate a sufficient number of inferences and consequently have difficulty establishing coherence (e.g., Cain & Oakhill, 2006), elaborators have difficulty building a coherent representation of text because of inappropriate use of background knowledge or personal viewpoints (e.g., Williams, 1993). The current study extended these previous findings by sketching a different picture for expository texts. For these texts, the low-comprehending elaborators obtained higher scores on the comprehension questions that elicited knowledge-based inferences. In other words, for expository texts the processing strategy of elaborators seems to have a positive influence on the quality of their mental representation.

The observation that elaborators obtained better mental representations for expository texts cannot be attributed to differences in decoding skills because the results from the tests battery suggest that in the current study the low-comprehending paraphrasers are in fact the better technical readers. However, the elaborators did outperform the paraphrasers on tests for inference and integration skills, receptive vocabulary knowledge, and verbal working memory. This could indicate that struggling paraphrasers do not yet possess all the abilities (cognitive or otherwise) required to generate inferences in order to comprehend expository texts at a deeper level. The lower scores of paraphrasers on receptive vocabulary knowledge, for instance, are an indication that they lacked the necessary background knowledge to generate accurate knowledge-based inferences, particularly in more demanding situations. In addition, low-comprehending paraphrasers may have been impeded by a smaller working-memory capacity. As a rule, reading expository texts imposes a heavier load on young readers’ working memory than reading narrative texts, because children tend to be less familiar with the structure and content of the text (Williams et al., 2004). In all, a tentative conclusion would be that working memory capacity and vocabulary knowledge are important precursors for generating accurate knowledge-based inferences, and that limitations in these cognitive domains hamper struggling paraphrasers in constructing a high-quality mental representations of expository texts.

Going beyond the analyses conducted in previous studies (McMaster et al., 2012; Rapp et al., 2007), we also explored whether subgroups can be distinguished for high-comprehending readers. We observed that, like low-comprehending readers, high-comprehending readers can also be categorized as paraphrasers and elaborators. These two types of high-comprehending readers performed equally well on the after-reading comprehension questions and all the tasks in the test battery. This shows that, for high-comprehending readers, multiple pathways (i.e., employing different reading strategies) produce the same level of comprehension (cf. McMaster et al., 2012).

### Text genre and discourse markers

A common assumption is that narrative and expository texts require (or at least elicit) different processing strategies due to differences in complexity and differences in readers’ familiarity with their structure (McDaniel & Einstein, 1989). An important aim of the current study was to explore whether young, unexperienced readers are already sensitive to the notion of genre, or whether they approach narrative and expository texts in a similar way. Our findings show that young children do indeed process narrative and expository texts differently. The think-aloud data revealed that in narrative texts the children more frequently explained the sentence they just read or generated a valid inference to obtain a deeper understanding of the text. In expository texts, the children experienced some problems during processing, as indicated by more (irrelevant) comments, more invalid inferences, and more silences. This was true for low-comprehending readers in particular, because as a group they generated significantly more inaccurate inferences for expository texts. These overall processing differences between narrative and expository texts also influenced the answers on the after-reading comprehension questions. It was more difficult for children to answer knowledge-based inference questions about expository texts than about narrative texts. Hence, it seems that what they were not able to do spontaneously during text processing, they were also not able to do when triggered by a question.

The good news is that sensitivity to text genre seems to apply to young low-comprehending readers as well as to young high-comprehending readers. The bad news is that most children, and in particular low-comprehending readers, do not possess all the tools to do what is required to understand expository texts. An important question, therefore, is how educators should provide children with these tools. One approach is to improve readers’ comprehension strategies. For example, McMaster et al. (2012, 2014) manipulated the reading task depending on the processing behavior of readers observed with regard to narrative texts: low-comprehending elaborators were stimulated to focus on important information within the text by answering causal questions during reading, whereas low-comprehending paraphrasers were stimulated to make text-connecting inferences and to think about the text beyond the current sentence by answering general questions during reading. A similar logic can be applied to improve young children’s comprehension of expository texts. It remains an open question however, what type of during-reading questioning is most effective in this case. On the one hand, it may be beneficial to develop questions for all young readers that ‘force’ them to stay close to the literal meaning of the text, because they seem to lack the relevant knowledge to generate accurate elaborate inferences. On the other hand, generating accurate inferences while reading expository texts is exactly the kind of skill that children should acquire at school, and hence educators should stimulate this. So, this raises the puzzle: How can we get the best of both worlds?

One way to address this puzzle is to encourage readers to pay special attention to discourse markers of a text such as connectives, referential pronouns, and markers representing topic organization. Discourse markers can be particularly helpful in expository texts because they guide readers’ comprehension of the relational and referential connections in a text, inducing (knowledge-based) inferences (cf. Land, 2009; Lorch, Lemarié, & Grant, 2011; Sanders, Land, & Mulder, 2007; van Silfhout, Evers-Vermeul, & Sanders, 2015). In other words, if young readers are encouraged to focus on discourse markers, they can be instructed to build their mental representation predominantly on the basis of the information provided by the text; at the same time, however, they are stimulated to connect different parts of the text, which promotes inferential processing.^3^

There is, however, another side to this coin. Although discourse markers can possibly prevent young (low-comprehending) readers from making invalid elaborative and predictive inferences, Best, Ozuru, Floyd, and McNamara (2006) have shown that young children benefit from coherence-marking in narrative texts, but not in expository texts. These authors suggest that in many expository texts discourse markers do not provide enough information for readers to make the necessary inferences without being provided with specific background information that is normally left unstated. Moreover, Land (2009) points out that although discourse markers can increase comprehension by adding cohesion, they also increase sentence length and complexity. In addition, discourse markers are abstract words and therefore difficult for less-skilled readers to comprehend.

### Implications for education

The current study provides important insights for education. First, the challenges that young, low-comprehending readers are facing are most likely of a ‘multimorbid’ nature. Second, both low- and high-comprehending readers can be classified as elaborators and paraphrasers. Third, both low- and high-comprehending readers are sensitive to text genre, but they do not possess the skills to adapt their processing strategy adequately. On the basis of these results, and inspired by the studies discussed throughout this contribution, we suggest multi-dimensional interventions. Where possible, these interventions should be custom-made by varying the instructions in light of the processing behavior of the child in combination with the type of text he or she is reading. Discourse markers may play an important facilitative role, especially in expository texts—although care should be taken that enough background knowledge is provided in the text (Best et al., 2006). Furthermore, in light of the results of the test battery and think-aloud data, all low-comprehending readers may benefit from pre-teaching of vocabulary, and from strategy instruction to interpret word meaning (Best et al., 2008; McMaster et al., 2014). Crucially, all interventions should take into account the limited working-memory capacity of low-comprehending readers (low-comprehending paraphrasers in particular). Finally, our study presents new insights to improve education for high-comprehending readers. Given that this group of readers also consists of elaborators and paraphrasers, they too may benefit from custom-made educational programs (cf. McMaster et al., 2012, 2014) to take their reading comprehension skills to the next level.

## Footnotes

1 We opted for children in Grade 2 because in the Netherlands formal education in reading comprehension and the use of reading strategies starts in Grade 3.

2 We used P-CLIB version 3.0 (Evers, 2008) to determine the difficulty level of the texts. The text difficulty was at *CLIB-4,* which equals Grade 2. Average text length was 146 words.

3 The experimental texts contained few discourse markers because the central interest concerned ‘unprompted’ text processing patterns to capture possible differences between low- and high-comprehending readers.
